# Editorial: Mechanism and therapy of autoimmune skin diseases

**DOI:** 10.3389/fmed.2023.1143454

**Published:** 2023-01-31

**Authors:** Xuming Mao, Jun Yamagami, Meng Pan

**Affiliations:** ^1^Department of Dermatology, University of Pennsylvania, Philadelphia, PA, United States; ^2^Department of Dermatology, Tokyo Women's Medical University, Tokyo, Japan; ^3^Department of Dermatology, Ruijin Hospital, School of Medicine, Shanghai Jiao Tong University, Shanghai, China

**Keywords:** autoimmune skin diseases, mechanisms, immunotherapies, manuscript submission, molecular technology

## Introduction

**Figure 1:**
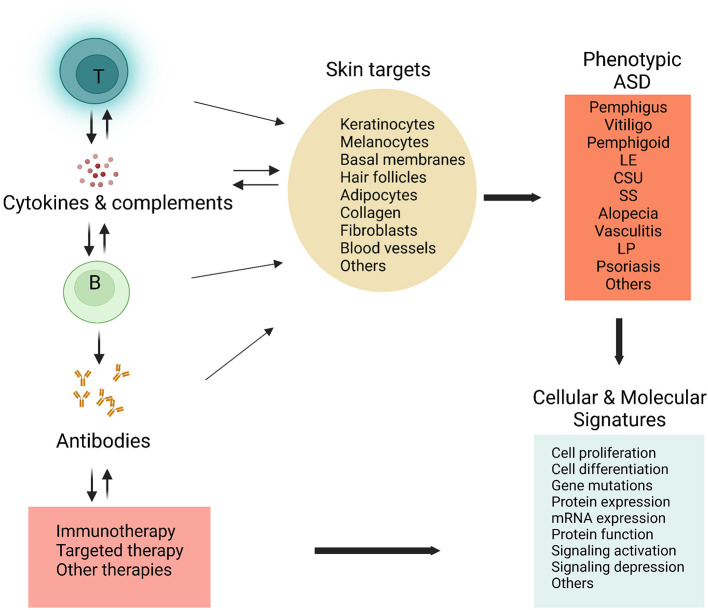
Schematic strategies in researching autoimmune or inflammatory skin diseases. Cellular or tissue components in the skin can be targeted by the immune effectors, including T lymphocytes (T), B lymphocytes (B), autoantibodies, and various cytokines. A broad range of cells or tissues are involved, leading to diverse skin diseases or ASD phenotypes. By the cellular and molecular approaches, such as microarray analysis, flow cytometry, immunofluorescence or immunohistochemistry, proteomics, metabolomics, and single-cell RNA sequencing, combined with state-of-art data analysis (bioinformatic analysis), characteristic cellular and molecular features or signatures could be identified in specific skin conditions. These findings may result in novel and more effective immunotherapies or targeted therapies for patients with ASD. ASD, autoimmune skin diseases; LE, Lupus erythematosus; LP, lichen planus; SS, systemic sclerosis; CSU, chronic spontaneous urticaria. The figure was created from Biorender (http://biorender.com).

It is a pleasure to know that the mission of a Research Topic of Frontier in Medicine, *Mechanism and therapy of autoimmune skin diseases*, has been accomplished. This Research Topic aims to recruit and publish novel research work that could advance our understanding or management of autoimmune skin diseases (ASD). In particular, the studies are supposed to focus on the mechanisms of how skin diseases are triggered, developed, and perpetuated in disease pathology. Based on the ASD pathophysiologies, novel targeted strategies with better efficacies could be developed for disease remission, control, or cure (Figure 1). As an exciting Research Topic, the issue has attracted inquiries for potential contributions and manuscripts from enthusiastic researchers or clinicians in many countries, including the United States, China, Germany, and Korea.

## The significance of studies on autoimmune skin diseases

The autoimmune disease remains to be a key public health concern across the world. More than 80 autoimmune diseases are known to be caused by autoimmunity, and have affected more than 25 million Americans (1) (National Health Institute). It is one of the top ten causes of death in women under the age of 65, and is the second highest cause of chronic illness (2). Notably, several commonly identified autoimmune diseases are skin-specific, such as psoriasis, vitiligo, scleroderma, and cutaneous lupus erythematosus (CLE), although other organs or systems could be involved. This type of disease has clearly been demonstrated to be a significant component of healthcare costs. Manuscripts on the current Research Topic comprise a variety of ASD, including psoriasis, lupus erythematosus (LE), pemphigus, vitiligo, lichen planus, bullous pemphigoid, and chronic spontaneous urticaria (CSU), which are intensively investigated Research Topics of the skin research.

## Distinct approaches were used to explore the mechanisms

New tools and technologies have significantly advanced our knowledge of human immunology and diseases (3). To elucidate the mechanisms of ASD, we are excited to find that a number of molecular and immunologic approaches were used in the studies. The goal is to identify the characteristic cellular and molecular signatures that are indispensable for developing precise diagnoses and specific treatments (Figure 1). In particular, microarray analysis and transcriptomic data have revealed the involvement of Th2 and Th17-related genes and pathways in CSU. In studying the role of B cell function in cutaneous LE, immunohistochemical analysis showed that lesional B cells form diffuse infiltrates or pseudofollicular structures, showing antigen-presenting and T cell-activating properties. In a cohort study, patients with adenotonsillar disease (ATD) were shown to have a significantly increased incidence of vitiligo, while surgical removal of the tonsils significantly lowered the rate of vitiligo in the patients. The study provided evidence for future studies to clarify the exact mechanism that induced autoimmunity in vitiligo.

## The challenge and opportunity for therapies for autoimmune skin diseases

Although traditional therapies have demonstrated potent efficacy, relapse is frequent during the long-term chronic disease courses of ASD. Combinational therapies may result in synergistic responses but can cause more adverse effects. Resistance to the therapies may develop, potentially due to loss of targets or immunophenotypic change. In addition, there are always interpatient variabilities that require optimization of the standard treatments. Although more and better therapeutic options are available for ASD, the management strategies for the chronic disorders remain to be further improved. As yet, the current mainstay therapy for most autoimmune diseases is systemic glucocorticoids and other immunosuppressants such as azathioprine (AZA), methotrexate (MTX), cyclosporine A (CSA), mycophenolate mofetil, and cyclophosphamide (CTX). However, these medications often lead to severe adverse effects, including infectious complications, hypertension, hyperglycemia, infertility, and an increased risk of cancers. Interestingly, an accepted manuscript presents the recent progress in the immunotherapies of pemphigus, an autoantibody-mediated ASD. Mechanism-based, novel immunotherapies appear promising in improving therapeutic efficacies and reducing side effects. Particularly, rituximab combined with short-term systemic glucocorticoids has been adapted as first-line therapy for treating pemphigus patients with excellent efficacies (4). Moreover, a cell-based chimeric antigen receptor T-cell (CAR-T) treatment has demonstrated excellent efficacy in treating pemphigus patients in pre-clinical studies (5). Collectively, the current evidence has supported that immunotherapies and targeted therapies are promising in managing recurrent or refractory ASD.

## The challenges for review and publication of review articles

This Research Topic prefers to publish original or novel results, although it accommodates diverse styles, including original articles, a case report and literature review, a clinical study, and review articles. The review articles summarize the previously published work. There are definitely tremendous values in this type of research, as they provide insights and identify the challenges of prior studies and potential future opportunities by reviewing the existing knowledge in the literature. The Research Topic will embrace novel ideas and experts' insights, as long as the review articles are relevant, comprehensive, and consistent. Indeed, a few submissions were accepted for publication, including one concerning the mechanisms and immune therapies for pemphigus, in which the authors overviewed the progress in the immune mechanisms and therapeutic agents used for therapies. Another review displays the therapeutic options of oral lichen planus (OLP). Indeed, erosions and ulcerations in OLP are notoriously refractory to standard therapies. The review highlighted the management options for oral LP and provided a preliminary clinical experience and insight that anti-IL17, anti-IL-12/IL-23, and anti-IL-23 monoclonal antibodies represent an effective and safe alternative therapy in refractory erosive/ulcerative OLP. Finally, the roles of immunoglobulin E (IgE) in comorbid psoriasis and atopic dermatitis AD are overviewed. This review shows that IgE significantly increased in a subset of psoriasis with AD-like presentations, suggesting that it plays a critical role in the pathogenesis of this type of skin condition. This study provides the rationale for a novel therapeutic target for the treatment of psoriasis comorbid with AD.

## Summary and conclusion

The current Research Topic has successfully drawn the attention of scientists in the ASD field in a short time frame. The potential contributions and submissions of manuscripts from researchers across the world presented exciting results and insights that are valuable in enhancing our knowledge in understanding the mechanisms and management, potentially significantly benefiting patients with ASD and physicians in the future.

## Author contributions

XM wrote the primary manuscript. XM, JY, and MP proposed and edited the manuscript. All authors contributed to the article and approved the submitted version.
